# Digital Choice Architecture in Medical Education: Applying Behavioral Economics to Online Learning Environments

**DOI:** 10.2196/86497

**Published:** 2026-02-06

**Authors:** Victoria Ekstrom

**Affiliations:** 1 Department of Gastroenterology and Hepatology, Singapore General Hospital, 20 College Road, Singapore, 169856, Singapore, 65 63214684; 2 Medical Humanities Institute, SingHealth Duke-NUS Academic Medical Centre, Singapore, Singapore

**Keywords:** medical education, behavioral economics, digital learning, learning management systems, LMS, choice architecture, learning analytics, nudge theory, implementation science

## Abstract

Health care has widely adopted behavioral economics to influence clinical practice, with documented success using defaults and social comparison feedback in electronic health records. However, online medical education, now the dominant modality for continuing professional development, remains designed on assumptions of rational learning that behavioral science has disproven in clinical contexts. This viewpoint examines the paradox of applying sophisticated behavioral insights to clinical work while designing digital learning environments as if learners are immune to cognitive limitations. We propose digital choice architecture for medical education: intentional integration of behavioral design principles into learning management systems and online platforms. Drawing from clinical nudge units and implementation science, we demonstrate how defaults, social norms, and commitment devices can be systematically applied to digital continuing education. As medical education becomes increasingly technology-mediated, behavioral science provides the theoretical foundation and practical tools for designing online learning environments that align with how clinicians actually make decisions.

## Introduction

Despite a global continuing medical education (CME) market projected to reach US $13.52 billion by 2028, continuing professional development (CPD) demonstrates remarkably limited effectiveness. A Cochrane systematic review of 215 studies with over 28,000 health care professionals found that educational meetings produce only a 6% median improvement in practice compliance and 3% improvement in patient outcomes [1]. Over 80% of CME activities rely on passive learning methods despite evidence showing that didactic presentations have little or no beneficial effect [2,3]. Health care organizations invest billions in educational technology while accepting single-digit effectiveness rates that would be unacceptable in any other clinical intervention.

The failure stems from flawed design assumptions. Current digital CPD platforms operate on an “information deficit model”—the assumption that providing knowledge changes behavior—which behavioral science has comprehensively debunked [2]. New information follows the Ebbinghaus forgetting curve, with 70% forgotten within 24 hours [3]. Physicians demonstrate poor self-assessment accuracy, with systematic reviews finding no correlation between self-assessed and measured competence in 65% of comparisons [4]. This exposes the paradox: we design learning platforms as if clinicians are perfectly rational decision-makers while deploying behavioral interventions in clinical systems because we know they are not.

Digital platforms now dominate CPD. In 2022, accredited providers reported 230,000 total CME activities—a 13% increase—with online modules representing the most common format [5]. However, despite this technological transformation, most digital medical education assumes that clinicians will engage systematically, complete modules through intrinsic motivation, and independently translate knowledge into practice change.

Behavioral economics challenges this assumption. In clinical settings, health care organizations have proven that physician behavior responds predictably to choice architecture. When Penn Medicine redesigned electronic health record (EHR) defaults to favor generic medications, prescribing rates increased from 75% to 98% without additional education [6]. Health care nudge units have demonstrated that defaults, social comparison feedback, and environmental cues systematically shape clinical decisions [7]. These interventions succeed because clinicians, despite extensive training, exhibit status quo bias, present bias, decision fatigue, and susceptibility to social norms [8,9]. Decision fatigue alone has measurable effects: physicians prescribe antibiotics more frequently as the day progresses [10].

This paradox demands resolution. As medical education investment accelerates toward digital delivery, designing platforms on disproven assumptions wastes resources and perpetuates the knowing-doing gap. The behavioral science infrastructure that transformed clinical practice—nudge units, implementation scientists, and A/B testing—remains largely absent from educational technology development. Closing this gap requires systematic integration of behavioral economics into digital learning architecture.

This viewpoint argues that digital medical education should evolve toward behaviorally informed design, creating online learning architectures that work with human cognitive realities. We propose comprehensive integration of behavioral economics principles into digital CPD, offering both theoretical foundations and practical implementation frameworks. It is important to note that while these principles have demonstrated effectiveness in clinical contexts, their educational applications remain emerging and context-dependent, requiring systematic evaluation across diverse learning environments.

## Terminology and Conceptual Clarity

Because behavioral economics terminology varies across disciplines, we establish the following definitions:

- Digital choice architecture refers to the systematic design of decision environments in digital learning platforms, encompassing defaults, interface structures, information displays, and feedback mechanisms that influence learner behavior while preserving choice. This term describes the overall design philosophy.
- Choice architecture represents the broader concept from behavioral economics, referring to how choices are organized and presented across any context [11].
- Behavioral design describes the practical application of behavioral economics and psychology principles to create systems supporting desired behaviors. We use this term when discussing the design process itself.
- Behavioral economics–informed design specifically indicates design decisions drawn from behavioral economics research, emphasizing the evidence-based grounding recommendations.
- Nudges are specific interventions that alter behavior predictably without forbidding options or significantly changing incentives [11]. Examples include defaults, social comparison feedback, and timely reminders.

These terms share conceptual overlap but maintain distinct emphases. Throughout this paper, we apply them consistently, distinguishing between broad design philosophy (choice architecture), practical implementation (behavioral design), specific interventions (nudges), and evidence-based approaches (behavioral economics–informed design).

## Theoretical Foundations: Core Behavioral Economics Concepts

Behavioral economics integrates psychology and economics to understand how people actually make decisions—often deviating predictably from rational actor models. For digital medical education, these insights may explain why well-designed platforms achieve dramatically different learning outcomes than behaviorally naive alternatives, though direct empirical evidence in educational contexts remains limited.

### Choice Architecture and Nudges

Choice architecture—defined by Thaler and Sunstein [11] as “organizing the context in which people make decisions”—recognizes that no choice presentation is neutral. Every interface element, default setting, and information display influences behavior. A comprehensive meta-analysis examining over 200 studies with 440+ effect sizes (N=2,148,439) found that choice architecture interventions promote behavior change with Cohen *d*=0.43, a small-to-medium effect practically significant for population-level interventions [8].

For digital learning platforms, choice architecture operates through every design decision. Whether modules appear in alphabetical order or by relevance to practice gaps shapes engagement. Whether completion tracking displays absolute progress (“3 of 10 modules”) or social comparison (“You’ve completed fewer modules than 67% of peers”) influences motivation through different behavioral mechanisms. The question is whether design reflects evidence or intuition.

Defaults and status quo bias leverage the tendency to stick with current states through multiple mechanisms: loss aversion, cognitive effort minimization, and implied endorsement [11]. Johnson and Goldstein [12] found that the organ donation consent rates were approximately twice as high in opt-out versus opt-in countries. Platforms that auto-enroll clinicians in modules addressing documented practice gaps leverage defaults while preserving opt-out capability.

Present bias and temporal discounting explain persistent procrastination. Clinicians understand CPD improves patient care, but immediate opportunity costs outweigh psychologically distant benefits. Laibson’s [13] quasi-hyperbolic discounting model demonstrates that people systematically prefer smaller immediate rewards over larger delayed rewards. On the basis of these findings, we propose that digital platforms may counteract this through temporal optimization—delivering microlearning at the point of clinical relevance, which minimizes the gap between learning effort and application reward.

Decision fatigue and cognitive depletion have measurable effects on clinical judgment. As noted, physicians prescribe antibiotics more frequently as the day progresses [10]. Judicial rulings decline from 65% favorable at session start to near zero before breaks, returning to baseline after rest [14]. For digital learning, this suggests that platforms should minimize unnecessary choices that deplete cognitive resources rather than exhausting capacity on navigation decisions.

Social norms and social comparison powerfully influence behavior. Social comparison feedback showing how behavior compares to peers consistently drives behavior change across clinical contexts. Digital dashboards displaying peer prescribing comparisons reduce inappropriate antibiotic use [15]. On the basis of the available evidence, we propose that learning dashboards could leverage similar mechanisms, though whether clinical effects translate to educational outcomes requires systematic investigation. Studies of collaborative learning modules show that embedding social cues could enhance motivation and learning clarity [16], suggesting that digital platforms could achieve similar benefits through discussion forums and peer-review features integrated into the learning management systems (LMSs).

### Integration With Complementary Theories

Behavioral economics does not operate in isolation. Cognitive load theory (CLT) provides mechanistic insights into working memory limitations—working memory can process only 4 to 7 information chunks simultaneously [17,18]. CLT distinguishes between intrinsic (inherent task difficulty), extraneous (imposed by suboptimal presentation), and germane (devoted to learning) cognitive loads. Choice architecture principles directly reduce extraneous cognitive load, freeing capacity for germane processing. The AMEE (Association for Medical Education in Europe) Guide No. 86 establishes CLT’s “particular relevance to medical education because many professional activities require simultaneous integration of multiple knowledge sets” [19].

Self-determination theory identifies 3 psychological needs essential for intrinsic motivation: autonomy, competence, and relatedness [20]. Williams et al [21] demonstrated that “autonomously motivated learning leads to better educational outcomes.” This creates productive tension with nudging approaches, as defaults can reduce perceived locus of causality. However, autonomy-preserving designs can reconcile these tensions through transparent nudges that explain rationale, self-nudges where learners choose their own interventions, and “boosts” that build competence rather than merely steering behavior. Hertwig and Grüne-Yanoff [17] distinguish boosting—which aims to “foster people’s competence to make their own choices”—from nudging, arguing that boosts create lasting cognitive “capital stock” and necessarily preserve autonomy.

These complementary frameworks converge on similar design principles. Behavioral economics explains why interventions work (cognitive biases and heuristics), CLT specifies how much information learners can process, and self-determination theory identifies what motivates sustained engagement.

## Digital Transformation and Clinical Lessons

The shift from classroom to technology-mediated education has fundamentally altered how clinicians access professional development. LMSs track every interaction—login times, completion rates, assessment attempts, and module abandonment points. Mobile apps deliver microlearning during clinical workflows. Artificial intelligence algorithms personalize content sequences. This digital infrastructure generates unprecedented behavioral data while creating new decision points where choice architecture can be applied. Every interface element implicitly shapes learning behavior. The question is not whether digital platforms incorporate choice architecture, but whether they do so intentionally and based on evidence.

Clinical decision support systems demonstrate what behaviorally informed digital design can achieve. EHRs now routinely incorporate default order sets favoring evidence-based practices. Digital dashboards provide peer comparison feedback on prescribing patterns. Predictive analytics trigger just-in-time reminders when clinical decisions deviate from best practice. A systematic review found robust evidence that digital defaults and social proof could meaningfully change a physician’s behavior across multiple domains [21]. Notably, these effects persist among experienced practitioners—behavioral tendencies are not eliminated by expertise. Digital choice architecture in clinical systems represents the convergence of behavioral science with technological capability, creating decision environments that systematically support better choices at scale.

## Behavioral Design Gap in Digital Medical Education

Applications of behavioral economics to online medical education exist but remain limited. Emerging evidence demonstrates both promise and important limitations.

Gurley et al [22] found that email reminders increased residency assessment completion rates, though the intervention required repeated prompts and the effect sizes were modest. More comprehensive interventions showed mixed results at scale. Kizilcec et al’s [23] landmark study tested 5 behavioral interventions across 269,169 students in 247 massive open online courses. Interventions showing effectiveness at a small scale (planning prompts: +29% completion for committed students) demonstrated minimal impact when implemented broadly. Only value-relevance interventions showed modest improvements for disadvantaged students in courses with existing achievement gaps. This large-scale evidence reveals critical constraints: behavioral interventions may require targeting and personalization, and what works in controlled trials may not translate to population-level deployment.

More promising results emerge from targeted interventions. A 2023 study combining learning analytics with behavioral nudges found personalized reminders significantly increased student resource clicks (planning courses: 415 to 778 clicks, *P*<.001; education courses: 304-446 clicks, *P*<.001) [24]. Research on tailored digital nudges matched to students’ motivation profiles showed improved engagement when interventions aligned with learner characteristics [25].

Medical education–specific applications remain rare. Van Gaalen et al’s [26] systematic review found that gamification in health professional education showed no negative outcomes, though most studies were descriptive with few using theory or well-defined control groups. Kerfoot et al’s [27] high-quality randomized controlled trial with 141 - medical students found that gamified and spaced learning improved hypertension management knowledge and produced modest reductions in days to reach target blood pressure—demonstrating transfer to patient outcomes.

Recent evidence from anatomy education demonstrated how behavioral principles could be successfully embedded. Specialized study modules (SSMs) incorporating intentional pathway design, collaborative learning structures, and clear learning objectives produced significant improvements in student motivation, teamwork, and clarity of learning goals [16,28]. These findings parallel mechanisms that digital choice architecture seeks to operationalize: both approaches rely on well-designed pathways that make desired behaviors easier, clearer, and more rewarding. Just as SSMs improved learning through structured guidance, digital platforms can leverage defaults, social nudges, and collaborative cues to streamline learning decisions and reduce friction. The observed heterogeneity in learning strategies across educational contexts [16] reinforces the need for adaptive nudges and personalized digital pathways accommodating diverse learner preferences.

Systematic reviews note that education-related studies represent only 4% of choice architecture interventions tested across all domains [29], with learning analytics in medical education “still in infancy” with “systematic applications limited” [30]. Platforms rarely use predictive analytics to identify at-risk learners or deploy targeted nudges. Research demonstrates that only half of people who form intentions actually take action [31], highlighting the critical intention-behavior gap. The majority of accredited CPD activities do not target clinical behavior change [32], with behavior change techniques appearing in only a subset of CME [32].

Considering typical design patterns in online CPD platforms, the default pathway after completing a module is returning to the dashboard—no implementation planning, no commitment to practice change, and no follow-up accountability. Courses are structured as optional selections requiring active search rather than defaults based on practice gaps. Progress tracking emphasizes completion percentages without peer comparison. Educational content is delivered months before clinical application, despite evidence that temporal distance undermines behavior change. The field that transformed clinical practice through digital behavior change has barely touched the digital systems used to educate clinicians.

## A Framework for Digital Choice Architecture in Online Medical Education

Digital choice architecture for medical education means intentionally designing online learning environments using behavioral principles proven effective in clinical contexts. We propose a comprehensive framework integrating established implementation science models with practical design principles. It is critical to emphasize that educational applications of these principles remain emerging, requiring context-specific adaptation and rigorous evaluation.

### Established Implementation Frameworks

Multiple frameworks can guide the systematic application of behavioral insights to educational platforms. The UK Behavioral Insights Team’s EAST (easy, attractive, social, and timely) framework provides the following accessible design heuristics [33] as : Make it Easy (reduce effort through defaults, simplified messages, and minimal “hassle factors”), Make it Attractive (use visual design, immediate rewards, and salient feedback), Make it Social (leverage peer comparison, network effects, and commitment to others), and Make it Timely (deliver prompts when learners are most receptive).

The COM-B (capability, opportunity, motivation, and behavior) model provides diagnostic capacity by conceptualizing behavior as arising from the interaction of 3 core components: capability (physical and psychological), opportunity (environmental and social enablers), and motivation (reflective and automatic processes) [34]. This diagnostic approach links to the behavior change wheel’s 9 intervention functions, enabling systematic selection of appropriate behavioral strategies based on identified barriers. The theoretical domains framework extends analysis across 14 granular domains, including knowledge, skills, professional identity, and beliefs about capabilities [35]. The Behavioral Insights Team’s 4-step method provides a cyclical process: define the outcome, understand the context, build your intervention, and test or learn or adapt [33].

### Stepwise Design Principles for LMS Platforms

Translating frameworks into practice requires systematic steps.

#### Step 1: Audit Current Choice Architecture

Before implementing behavioral design, organizations must document existing implicit architecture. This includes mapping all default settings. For example, what happens when learners complete a module? What enrollment pathways exist? Organizations should identify every decision point where learners make choices, catalog implicit assumptions embedded in platform design, and establish baseline behavioral metrics including completion rates, time-to-completion, drop-off points, and peak engagement times. This audit reveals where behavioral insights can add value while providing baseline data for measuring improvement.

#### Step 2: Apply Behavioral Design Principles

Defaults leverage status quo bias. Auto-enrollment in required modules based on practice type or documented knowledge gaps preserves choice while making evidence-based learning the path of least resistance. Research demonstrates that providing clear, guided pathways—similar to defaults—improves motivation and learning clarity while reducing cognitive burden [16,28]. Evidence-based pathway before selection prioritizes high-impact content. Postmodule defaults should route learners to implementation planning tools rather than simply returning to catalogs. Assessment scheduling can prepopulate optimal spacing intervals. Each default preserves learner autonomy through transparent opt-out.

Friction reduction addresses that every additional click creates abandonment opportunity. One-click enrollment minimizes barriers. Single sign-on eliminates authentication friction. Streamlined navigation reduces unnecessary steps. Form simplification eliminates nonessential fields. These micro-optimizations cumulatively reduce extraneous cognitive load.

Social comparison and peer feedback leverage descriptive norms without public shaming. Learning dashboards can display “You have completed 60% of required CPD; 80% of peers in your specialty have completed more.” Anonymized leaderboards show rankings for voluntary activities. Specialty-specific benchmarks compare engagement to relevant peer groups. Completion milestones highlight social norms: “Join the 73% of cardiologists who completed this module.” Peer-supported learning structures enhance motivation and learning clarity [16], suggesting that platforms can achieve benefits through discussion forums, peer-review features, and collaborative assignments integrated into LMSs.

Timely prompts and momentum nudges address present bias. Deadline reminders sent 3 days before, 1 day before, and on the day counteract procrastination. Momentum messages like “You’re 80% through this module—finish now?” capitalize on progress. Peak engagement timing delivers notifications at moments when individual users are most likely to engage, based on their historical behavior. Calendar integration schedules protected learning time directly into clinicians’ calendars.

Personalization based on behavioral data increases relevance while reducing cognitive load. Role-based recommendations curate content by specialty. Practice-gap-informed suggestions link learning to quality metrics. Adaptive difficulty adjusts complexity based on assessment performance. Temporal optimization delivers notifications when individual learners historically show the highest engagement.

#### Step 3: Implement With Transparency

Ethical behavioral design requires disclosure and opt-out mechanisms. Platform documentation should clearly explain that the system uses behavioral design principles. Opt-out mechanisms must allow users to disable social comparison features, modify notification frequency, or change default pathways. Transparent nudges make intent reconstructable—for example, “This module was recommended based on your practice gaps in diabetes management.” All behavioral design choices should be documented and reviewed through appropriate oversight mechanisms. Research shows transparent nudges remain effective [36,37].

#### Step 4: Measure and Iterate

Continuous measurement enables data-driven improvement. Organizations should track engagement metrics (logins, completion rates, time-on-platform, and drop-off points), learning outcomes (assessment scores and knowledge retention), and practice change where feasible (quality metrics and chart audits). A/B testing allows systematic comparison of behavioral interventions. User feedback through surveys and qualitative interviews provides insight into learner experience. On the basis of these data, organizations should refine interventions quarterly, abandoning approaches that do not work and scaling those that do.

#### Digital Commitment Devices and Implementation Intentions

The intention-behavior gap represents a critical barrier. Research demonstrates that only half of the people who form intentions actually take action [31]. Digital platforms can incorporate commitment mechanisms grounded in implementation intention research, which shows medium-to-large effect sizes (Cohen *d*=0.65) when individuals create specific if-then plans [38]. Practical applications include implementation planning prompts after module completion requiring learners to specify “When will you apply this? In what clinical situation?” Calendar commitments directly schedule follow-up learning or application time blocks. Automated accountability check-ins follow up, asking “Did you apply what you learned? What barriers did you encounter?”

## Implementation Roadmap for Health Care Organizations

Health care organizations can follow these phased approaches.

### Phase 1: Assessment and Planning (Months 1-3)

Phase 1 includes the following:

1. Learning analytics specialists conduct comprehensive choice architecture audits documenting all platform defaults, decision points, and implicit assumptions.
2. Learner surveys identify motivation barriers and preferences while behavioral analytics reveal completion patterns and peak engagement times.
3. Deliverables include current state reports, priority intervention lists ranked by impact-effort ratio, baseline metrics dashboards, and stakeholder engagement plans. This requires a learning analytics specialist, behavioral scientist consultant (0.2‐0.5 full-time equivalents), platform administrator, end-user clinicians, and an instructional designer.

### Phase 2: Pilot Interventions (Months 4-9)

Phase 2 consists of the following:

1. Implement 3 to 5 behavioral design changes in a limited scope, typically targeting a single department.
2. Conduct A/B testing where feasible. Measurement includes quantitative metrics (completion rates, time-to-completion, and assessment performance) and qualitative methods (user feedback and focus groups). Example pilots: changing default postmodule action to “create implementation plan,” adding peer comparison feedback to learner dashboards, implementing smart reminders based on individual engagement patterns, testing auto-enrollment versus opt-in, or adding commitment devices.
3. Track completion rates, time-to-completion, assessment performance, user satisfaction, opt-out rates, and qualitative feedback themes.

### Phase 3: Scale and Sustain (Months 10+)

Phase 3 consists of the following:

1. Roll out successful interventions system-wide.
2. Build ongoing A/B testing infrastructure.
3. Train staff in behavioral design principles.
4. Establish governance structures for ethical oversight.
5. Integrate behavioral metrics into routine quality improvement cycles.
6. Share results through publications and presentations.
7. Sustainability requires behavioral science expertise (0.5‐1.0 FTE), learning analytics infrastructure, regular review cycles, ethical oversight committees, and continuous professional development.

### Resource Requirements and Common Pitfalls

Resource requirements scale with organizational size. Small organizations serving fewer than 5000 learners typically invest US $50,000 to US $100,000 annually, including part-time behavioral science consultation, analytics platform subscriptions, and platform customization. Medium organizations serving 5000 to 50,000 learners typically invest US $200,000 to US $400,000 annually, including full-time behavioral science expertise, dedicated analytics platforms, testing infrastructure, and platform development. Large organizations serving over 50,000 learners typically invest US $500,000 to US $1 million or more annually, including behavioral science teams, advanced analytics platforms, dedicated nudge unit infrastructure, and platform development teams. These figures represent ongoing investments necessary for effective educational delivery in the digital age.

Organizations should avoid common implementation pitfalls. Overcomplication from starting with too many simultaneous interventions makes isolating effects impossible; organizations should begin with 2 to 3 high-priority changes, demonstrate value, and then expand. Metrics myopia from optimizing purely for completion rates without monitoring quality or satisfaction can lead to gaming behaviors; use balanced scorecards instead. Transparency failures from implementing behavioral interventions without disclosure violate ethical principles and risk backlash; always explain design rationale up front. Ethical shortcuts from rushing implementation without ethical review create institutional risks; establish oversight early. Siloed work with behavioral science operating independently from instructional design creates a fragmented user experience; integrate from the start. Technology determinism assumes that building features automatically change behavior without attention to psychology; focus on behavioral science first and technology second. Insufficient testing by scaling interventions before demonstrating effectiveness wastes resources; pilot rigorously before scaling. Ignoring heterogeneity by assuming all learners respond identically misses personalization opportunities; conduct segment analyses and adapt interventions accordingly.

## Research Agenda

Translating behavioral economics from clinical to educational digital systems requires systematic investigation. Key priorities include conducting comprehensive audits of existing online CPD platforms to document current choice architecture, cataloging defaults and behavioral assumptions. Randomized controlled trials should compare traditional digital learning designs with behaviorally optimized alternatives, measuring not just completion but practice change outcomes. Implementation frameworks need development to provide practical guidance for educational institutions.

The key research questions are as follows:

1. Do behavioral interventions remain effective in clinical EHRs transfer to educational LMS contexts with similar effect sizes?
2. How does professional identity moderate responses to digital educational nudges?
3. What is the optimal balance between algorithmic personalization and learner autonomy?
4. How do clinicians respond to transparent disclosure that platforms incorporate behavioral design?

Technical implementation requires interdisciplinary collaboration [39]. Educational technologists must partner with behavioral scientists to translate abstract principles into specific interface elements. Data scientists need frameworks for analyzing learning analytics through a behavioral lens. Software developers require training in ethical behavioral design to avoid dark patterns that maximize compliance at the expense of learning quality or user autonomy.

## Ethical Implications of Behavioral Design in Digital Learning

Applying behavioral economics to digital education raises legitimate concerns about manipulation and autonomy. Well-designed nudges can preserve freedom of choice while supporting goal achievement [40]. The distinction between facilitation and manipulation lies in transparency, alignment with learner objectives, and preservation of autonomy.

### The Hansen and Jespersen Framework

Hansen and Jespersen [36] provide essential ethical distinctions through a 2D typology. Nudges vary along cognitive processing (type 1 automatic vs type 2 reflective) and transparency (transparent vs nontransparent intent and mechanism). Transparent type 2 nudges engage reflective processing while making intent and mechanism clear—these “do not comprise the unethical aspect of manipulating or secretly influencing individuals’ behavior” and represent the gold standard [37]. For example, “This module was recommended based on your documented practice gaps in diabetes management. View criteria and opt out if not relevant.” Transparent type 1 nudges operate through automatic processing but are disclosed. For example, “We’ve pre-selected the most commonly chosen learning path for your specialty. You can customize at any time.” Nontransparent nudges raise manipulation concerns and are generally impermissible in educational contexts.

### Supportive Versus Coercive Design

Supportive nudges preserve choice through easy, clear opt-out mechanisms. They maintain transparent intent, align with learners’ stated goals, build capability alongside behavioral steering, and respect autonomy. In contrast, coercive manipulation obscures alternatives or makes opt-out difficult. It uses hidden intent, optimizes for platform engagement metrics rather than learner welfare, and uses dark patterns such as fake urgency, hidden costs, forced continuity, or obstruction.

Research decisively shows that transparent nudges are not less effective than nontransparent alternatives [36,37]. This evidence suggests that transparency serves as an “ethical filter” without sacrificing outcomes, meaning platforms can—and should—openly explain their behavioral design principles while maintaining effectiveness.

### Ethical Decision Framework: 5-Step Process

Organizations implementing digital choice architecture should apply the following framework before deploying any intervention:

1. Is the nudge transparent? Can learners reconstruct the intent?
2. Does it engage reflective processing (type 2)? Prefer interventions that prompt conscious consideration.
3. Is opt-out easy and clear? Can learners disable the intervention in 3 clicks or fewer?
4. Does it align with learner objectives? Is the intervention designed to help learners achieve their stated goals?
5. Does it build competence alongside behavior change? Does the intervention include educational components explaining why the behavior is beneficial?

If an intervention fails any criteria, it should be redesigned or rejected.

### Dark Patterns: Explicitly Prohibited

Educational platforms must avoid deceptive design practices including fake scarcity (“Only 3 spots left!” when capacity is unlimited), hidden costs, forced continuity, obstruction (making cancelation unreasonably difficult), misdirection, nagging (excessive notifications), and sneaking (adding items without consent). These practices violate trust, undermine autonomy, and have no place in professional education.

## The Future of Behaviorally Informed Digital Medical Education

The integration of behavioral economics into digital medical education represents a paradigm shift from information-deficit models to evidence-based decision support. As artificial intelligence and adaptive learning technologies mature, behavioral design principles will become increasingly critical. Machine learning algorithms can personalize nudges based on individual response patterns, learning analytics can identify optimal timing for interventions, and natural language processing can generate personalized feedback. However, technological sophistication must be matched by ethical sophistication. The more powerful these tools become, the more important transparency and oversight become.

Looking forward, digital choice architecture may extend beyond traditional CPD to include just-in-time clinical decision support that simultaneously educates and guides practice, simulation-based training environments that use defaults and social comparison to accelerate skill acquisition, and longitudinal professional development pathways that adapt to career stages and practice evolution. The convergence of behavioral science, educational technology, and implementation science offers unprecedented opportunity to close the knowing-doing gap that has plagued medical education for decades.

## Limitations and Future Research

This viewpoint has important limitations. First, we extrapolate from clinical behavioral interventions to educational contexts without comprehensive evidence that effect sizes transfer. Educational effects of behavioral design principles are inferred from clinical contexts and remain context-dependent, requiring systematic empirical validation across diverse learning environments before widespread adoption. The Kizilcec et al’s [23] study demonstrates that small-scale educational successes may not replicate at the population level, suggesting caution in assuming that clinical behavioral economics directly translates to educational platforms. Second, we focus primarily on CPD rather than undergraduate or postgraduate training, where different considerations may apply. Third, ethical frameworks proposed require empirical validation—we need evidence on how transparent nudges affect trust, engagement, and learning outcomes specifically in medical education contexts.

Fourth, there exists a risk of overoptimization or technocratic control, where excessive focus on measurable behavioral metrics (completion rates and click-through rates) may overshadow harder-to-quantify educational outcomes such as critical thinking, professional identity formation, or intrinsic motivation. Behavioral design could inadvertently create systems that optimize for compliance rather than meaningful learning.

Fifth, resistance from clinicians who value professional autonomy represents a significant implementation barrier. Physicians may perceive behavioral interventions—even transparent ones—as patronizing or as threats to professional independence. This resistance may be particularly strong among senior clinicians with established practice patterns who view educational decisions as matters of professional judgment rather than targets for behavioral modification.

Sixth, institutional constraints pose substantial challenges. These include cost barriers (significant upfront investment in behavioral science expertise, analytics infrastructure, and platform redesign), governance complexities (establishing ethical oversight, determining acceptable intervention boundaries, and managing stakeholder concerns), and legacy system limitations (many organizations operate on established LMS platforms with limited customization capabilities, vendor dependencies, and lengthy procurement cycles that impede rapid iteration). Health care organizations must navigate these practical constraints while attempting implementation.

Future research should prioritize large-scale randomized trials comparing behaviorally optimized platforms to traditional designs across multiple outcomes (completion, learning, satisfaction, and practice change). Studies should examine heterogeneity in treatment effects—Which clinician subgroups respond most strongly to which interventions? Implementation science research should document barriers and facilitators to adopting behavioral design in health care organizations. Ethical research should examine learner perceptions of nudges, optimal disclosure methods, and long-term effects on professional development autonomy.

## Conclusions

The question facing medical education is not whether to design learning environments but whether to design them well or badly. Every digital interface embodies choice architecture—defaults, menu structures, notification timing, and progress displays—that shapes learning behavior. Currently, most platforms incorporate architecture based on intuition rather than evidence. This represents a missed opportunity of staggering proportions.

Health care organizations should conduct choice architecture audits of existing CPD platforms, identifying defaults and decision points; pilot behavioral interventions with rigorous measurement before scaling; and partner with behavioral scientists during platform design, not as an afterthought. Academic medical centers should deploy implementation science frameworks to systematically translate behavioral insights into educational practice. Accreditation bodies should incentivize behavioral design through quality metrics that reward practice change, not just completion rates. Vendors should partner with behavioral scientists from the design stage, building behavioral principles into product architecture. Professional societies should develop best practice guidelines for ethical behavioral design in medical education, facilitate sharing through communities of practice, and advocate for research funding.

The evidence base exists. The tools are available. The imperative is urgent. The path is clear. The question is whether we will take it.

## Abbreviations

AMEE: Association for Medical Education in Europe
CLT: cognitive load theory
CME: continuing medical education
COM-B: capability, opportunity, motivation, and behavior
CPD: continuing professional development
EAST: easy, attractive, social, and timely
EHR: electronic health record
LMS: learning management system
